# The influence of chronic diseases and multimorbidity on entering paid employment among unemployed persons – a longitudinal register-based study

**DOI:** 10.5271/sjweh.3942

**Published:** 2021-03-31

**Authors:** Berivan Yildiz, Alex Burdorf, Merel Schuring

**Affiliations:** Department of Public Health, Erasmus University Medical Center, Rotterdam, The Netherlands

**Keywords:** PAF, population attributable fraction, register data, medicine, unemployment

## Abstract

**Objectives:**

This study aimed to investigate the influence of chronic diseases and multimorbidity on entering paid employment among unemployed persons. A secondary objective was to estimate the proportion of persons not entering paid employment that can be attributed to specific chronic diseases across different age groups.

**Methods:**

Data linkage of longitudinal nationwide registries on employment status, medication use and socio­demographic characteristics was applied. Unemployed Dutch persons (N=619 968) were selected for a three-year prospective study. Cox proportional hazards analyses with hazard ratios (HR) were used to investigate the influence of six common chronic diseases on entering paid employment, stratified by age. The population attributable fraction (PAF) was calculated as the proportion of all persons who did not enter paid employment that can be attributed to a chronic disease.

**Results:**

Persons with chronic diseases were less likely to enter paid employment among all age groups. The impact of a chronic disease on maintaining unemployment at population level was largest for common mental disorders (PAF 0.20), due to a high prevalence of common mental disorders (6%), and for psychotic disorders (PAF 0.19), due to a high likelihood of not entering paid employment (HR 0.21), among persons aged 45–55 years. Multimorbidity increased with age, and the impact of having multiple chronic diseases on remaining unemployed increased especially among persons aged ≥45 years.

**Conclusion:**

Chronic diseases and multimorbidity are important factors that reduce employment chances among all age groups. Our results provide directions for policy measures to target specific age and disease groups of unemployed persons in order to improve employment opportunities.

Employment status is an important determinant of health, as demonstrated by a lower mental and physical health status among unemployed persons (1). These health inequalities can be explained by the causation and selection hypothesis. The causation hypothesis states that unemployment causes persons to deteriorate in health, whereas employment causes persons to improve in health. The selection hypothesis states that persons with poor health have a higher risk of leaving paid employment, as well as remaining unemployed for a longer period (2, 3).

Many studies have shown that chronic diseases, such as psychological disorders, cardiovascular diseases and diabetes, are typically more common among unemployed individuals (4–7) and are associated with a higher risk of being unemployed (8). However, most studies address loss of paid employment in the workforce rather than health as a barrier for (re)entering paid employment (9). Many qualitative studies have shown that entering paid employment is often challenging for persons with mental health problems (10, 11). Some quantitative studies also have shown that persons with psychological or physical health conditions have a lower likelihood of entering paid employment (12–15). Less is known about the influence of having a specific chronic disease or multiple chronic diseases on entering paid employment. Especially those who have multiple chronic diseases seem to be less likely to enter paid employment. In fact, multimorbidity – the occurrence of at least two chronic diseases within an individual – has been associated with a higher risk of unemployment (16). One study showed that unemployed individuals with two health conditions had a 24% decreased likelihood of entering paid employment compared to those with no health conditions (14).

So far, most studies on associations between chronic diseases and entering paid employment have mainly focused on self-reported health outcomes and some have focused on certain health aspects such as mental health only (6, 17). However, self-reported health outcomes are prone to reporting bias and justification bias. This often occurs when unemployed individuals over-report their level of disability or work limitations to justify that they are not in paid employment (18). Therefore, in order to minimize bias, it is important to use more objective data. One way to objectively investigate the presence of chronic diseases is by using pharmacy data (19). Pharmacy data on dispensed drugs provide a reliable information source, often covering a broad range of health aspects in the general population (20). Linkage of objective pharmacy data with employment registers can create large study populations that facilitate precise estimations of associations between health and employment.

Only few studies have used pharmacy data as a health indicator in relation to entering employment. One Danish study found that prescription medicine purchases for both physical illnesses (including cardiovascular diseases, chronic lung diseases and type 2 diabetes) and mental illnesses had a negative influence on the likelihood of entering paid employment (21). Another study showed that prescribed medication purchases for mental illnesses significantly reduced employment chances in a random population sample (22). However, these studies have focused on single chronic diseases and their associations with entering paid employment rather than the impact of an array of (multiple) chronic diseases on entering paid employment at population level. In addition, there is little insight in the age-specific influence of chronic diseases on entering paid employment. The sheer size of the present study facilitates to study multimorbidity and age-specific patterns in the impact of chronic diseases on employment, which is a unique feature of this study. This study does also not suffer from any selection bias into the study population as the total Dutch population of unemployed persons was included.

Age is closely related to the presence of chronic diseases as well as the likelihood of entering paid employment (14, 23, 24). In general, older persons often encounter more barriers in the labor market than younger persons, resulting in a lower likelihood of entering paid employment (25, 26). Chronic diseases may impact individuals differently according to the phase in their working life course. For instance, younger persons are more likely to aim for a successful career compared to older persons who are almost at the end their careers (27). Therefore, diseases may have a larger impact on employment among younger compared to older persons.

However, little is known about how age and chronic diseases may interact to create or increase barriers to enter paid employment. Therefore, the influence of chronic diseases on entering paid employment should ideally be explored among different age groups. It is of particular interest to know how the influence of specific chronic diseases on paid employment varies across disease groups and whether the presence of multiple chronic diseases has synergistic or independent effects. This is especially of high importance for interventions targeting health and improving employment opportunities for unemployed persons.

Most longitudinal studies have focused on associations of chronic diseases with entering paid employment, interpreting these associations at individual level, ie, the reduced risk for an individual person with a chronic disease to enter or maintain paid employment. This information can only be generalized to the total population, when the study population is a random sample of the total population. A population-based approach offers the opportunity to estimate the contribution of chronic diseases to being without paid employment in the population. In this sense, the population attributable fraction (PAF) is the proportion of persons without paid employment that can be attributed to the presence of chronic diseases. The PAF is an integrated measure that takes into account both the strength of association between the risk factor and the outcome as well as the prevalence of the risk factor in the population. This measure reflects the theoretical reduction in unemployment in the population that would occur if the prevalence of chronic diseases were reduced to zero, or chronic diseases no longer had any effect on likelihood of paid employment.

In the present study, nationwide data on employment status, medication, and sociodemographic characteristics were used to conduct a longitudinal study with a three-year follow-up. The primary aim of the study was to investigate the influence of chronic diseases and multimorbidity on entering paid among unemployed persons. The secondary aim was to estimate the proportion of persons not entering paid employment that can be attributed to specific chronic diseases across different age groups.

## Methods

### Study design and population

A longitudinal study in The Netherlands with a three-year follow-up was conducted on register data covering information on residents between January 2015 and December 2017. Statistics Netherlands provided secure access to individual-level databases on demographics, educational level, labor market status, and dispensed medication. All Dutch residents were pseudonymized using a personal unique number. Data registries were linked at the individual level using these pseudonymized numbers. In the present study, persons who received social security benefits or unemployment benefits on 1 January 2015 (baseline), were not employed for at least the previous three months, and were aged 18–65 years (N=625 691) were selected. No informed consent was needed to conduct this study as Dutch legislation allows authorized research institutes to use pseudonymized register-based data for research purposes. To report the findings of this study, the STROBE guidelines were followed.

### Employment status

The database on socioeconomic position per month (SECM) provided the main source of income for each consecutive month between 2014 and 2017. Persons who received a social security or unemployment benefit were defined as unemployed. Persons who were employees or self-employed were classified as employed. In this longitudinal study, the transition from unemployment into paid employment was investigated.

### Chronic diseases and multimorbidity

The database on medication (Medicijntab) provided information on purchased drugs that were reimbursed by health insurance companies. Since no data was available on the precise dates of the drugs being purchased during the year, medication use in 2014 was used to identify the presence of chronic diseases among unemployed persons (19), based on the World Health Organization Anatomical Therapeutic Chemical (ATC) classification codes (28). For instance, common mental disorders were identified by ATC codes assigned to drugs such as antidepressants and anxiolytics, whereas respiratory illness was identified by the ATC code assigned to inhalants and other drugs for airway diseases. The presence of a specific chronic disease was dichotomized into having or not having a chronic disease. The most prevalent chronic diseases investigated in this study were: inflammatory conditions, common mental disorders, psychotic disorders, cardiovascular diseases, diabetes, and respiratory illness (supplementary material, www.sjweh.fi/show_abstract.php?abstract_id=3942, table S4).

Multimorbidity was investigated as the number of chronic diseases. The total number of chronic diseases was computed for each participant, based on the 21 different chronic diseases identified by medication. This measure of multimorbidity was then categorized into four groups: 0, 1, 2, ≥3 chronic diseases. Having ≥2 chronic diseases was defined as multimorbidity.

### Sociodemographic variables

The databases of Statistics Netherlands on sociodemographic characteristics provided information on age, sex, education, and migration background in 2014. Educational level was categorized into three educational groups: high (higher vocational training or university), intermediate (higher secondary and intermediate vocational training) and low education (pre-primary education, primary education, and lower secondary education). Age was categorized into four age groups (18–30, 30–45, 45–55, 55–65 years). Migration background was categorized into six groups: native Dutch, Moroccan, Turkish, Surinamese and Antillean, other Western, other non-Western.

### Analyses

Descriptive statistics were used to describe baseline characteristics. Cox proportional hazards analyses were performed to investigate the influence of each chronic disease on entering paid employment. The dependent variable was the first event of entering paid employment for at least three consecutive months during the three-year follow-up period. Persons were censored at the transition from unemployment to disability or (early) pension, after three months of missing data on employment status, and at the end of the follow-up period (December 2017). The same was done to investigate the influence of multimorbidity on entering paid employment. Stratified analyses were performed to estimate associations within age groups. All analyses were adjusted for sex, educational level and migration background.

Since this study was conducted on all unemployed persons in The Netherlands, the discriminatory power is high and traditional testing of statistical significance has little relevance as we do not have a sample but the entire population. Hence, we present confidence intervals (CI) but refrain from formal statistical tests when deemed not informative.

In order to investigate whether the effect estimates were significantly different between the four age groups, an interaction term of chronic disease×age was added to the cox proportional hazards models on the non-stratified population (N=619 968). An interaction term of multimorbidity×age was also included in the Cox proportional hazards analysis.

The PAF can be defined as the proportion of unfavorable outcomes (remaining unemployed) that would have been prevented if the exposure of interest (chronic diseases) was eliminated from the population. The following formula was used: PAF=Pe(HR-1)/(1+Pe(HR-1), where Pe is the prevalence of a chronic disease among a particular age group (29). The hazard ratio (HR) in this formula is the likelihood to remain unemployed, which is the inverse of the HR to enter paid employment. A PAF close to 1 indicates that remaining unemployed is almost completely attributed to the chronic disease. A PAF close to 0 indicates that chronic diseases do not play a role in remaining unemployed. The PAF was also calculated at each age between 25 and 60 years, based on age-specific prevalence and HR. All statistical analyses were performed using SPSS v.22.0 (IBM Corp, Armonk, NY, USA).

## Results

Approximately half of the unemployed persons were older than 45 years (45.9%), female (52.2%), lower educated (45.1%), and native Dutch (54.3%). The most prevalent chronic diseases were inflammatory conditions (24.8%), cardiovascular diseases (18.2%), and common mental disorders (17.3%). Almost 60% of the study population had at least one chronic disease. The prevalence of having at least one chronic disease was higher among unemployed persons (59.1%) compared to employed persons (39.8%) in The Netherlands (table 1, and supplementary table S1)

**Table 1:** Baseline characteristics of the study population (N=619 968).

	N (%)
	
Age (years)	
18–30	72 884 (11.8)
30–45	200 829 (32.4)
45–55	175 236 (28.3)
55–65	171 019 (27.6)
Sex	
Male	296 515 (47.8)
Female	323 453 (52.2)
Educational level	
High	92 150 (14.9)
Middle	213 501 (34.4)
Low	279 782 (45.1)
Missing	34 535 (5.6)
Migration background	
Native Dutch	336 916 (54.3)
Moroccan	40 686 (6.6)
Turkish	32 363 (5.2)
Surinamese & Antillean	49 221 (7.9)
Other Western	48 111 (7.8)
Other non-Western	112 671 (18.2)
Chronic conditions	
Inflammatory conditions	153 815 (24.8)
Cardiovascular diseases	112 987 (18.2)
Common mental disorders	107 557 (17.3)
Respiratory illness	67 403 (10.9)
Diabetes	40 227 (6.5)
Psychotic disorders	33 775 (5.4)
Multimorbidity	
No chronic diseases	253 471 (40.9)
One chronic disease	147 994 (23.9)
Two chronic diseases	93204 (15.0)
Three or more chronic diseases	125 299 (20.2)

The proportion of persons entering paid employment during the follow-up period was highest for persons aged 18–30 years (46.0%), followed by the age groups 30–45 (39.8%), 45–55 (33.3%), and 55–65 (15.3%) years. The likelihood to enter paid employment reduced with increasing age. The likelihood to enter paid employment also reduced with increasing number of chronic diseases. The prevalence of multimorbidity was highest among the older age groups, with the highest prevalence of ≥3 chronic diseases (30.9%). Persons with ≥3 chronic diseases had the lowest likelihood to enter paid employment, with the lowest likelihood among persons aged 30–45 years (HR 0.43, 95% CI 0.42–0.44). The presence of multiple chronic diseases showed no synergistic effects on entering paid employment, indicating chronic diseases to be largely independent of each other. The fraction of persons staying unemployed due to having at least three chronic diseases increased from 0.04 in the youngest age group to 0.22 in the oldest age group (table 2, supplementary table S2)

**Table 2a:** The prevalence of multimorbidity and the association with entering paid employment among unemployed persons, stratified by age (N=619 968).

	18–30 (N=72 884)			30–45 (N=200 829)		
	
	%	HR (95% CI)	PAF (95% CI)	%	HR (95% CI)	PAF (95% CI)
Enter employment	46.0			39.8		
Chronic diseases						
0	58.0	1		47.1	1	
1	25.6	0.83 (0.81–0.85)	0.02 (0.02–0.03)	26.0	0.83 (0.82–0.84) ^a^	0.05 (0.04–0.05)
2	10.4	0.67 (0.64–0.70)	0.04 (0.03–0.04)	13.9	0.64 (0.63–0.66) ^a^	0.07 (0.07–0.08)
≥3	6.0	0.55 (0.52–0.58)	0.04 (0.03–0.04)	12.9	0.43 (0.42–0.44) ^a^	0.15 (0.14–0.15)

a Significant difference between age groups (p<0.005), reference group: persons aged 18–30 years.

**Table 2b:** The prevalence of multimorbidity and the association with entering paid employment among unemployed persons, stratified by age (N=619 968).

	45–55 (N=175 236)			55–65 (N=171 019)		
	
	%	HR (95% CI)	PAF (95% CI)	%	HR (95% CI)	PAF (95% CI)
Enter employment	33.3			15.3		
Chronic diseases					
0	36.6	1		30.6	1	
1	23.2	0.87 (0.85–0.89) ^a^	0.04 (0.03–0.04)	21.2	0.93 (0.91–0.97)	0.01 (0.01–0.02)
2	16.1	0.70 (0.69–0.72) ^a^	0.07 (0.06–0.07)	17.2	0.78 (0.75–0.81)	0.05 (0.04–0.06)
≥3	24.0	0.44 (0.42–0.45) ^a^	0.24 (0.23–0.25)	30.9	0.52 (0.50–0.54) ^a^	0.22 (0.21–0.23)

a Significant difference between age groups (p<0.005), reference group: persons aged 18–30 years.

Persons with common mental disorders, psychotic disorders, cardiovascular diseases, inflammatory conditions, diabetes and respiratory illness were all less likely to enter paid employment among all age groups. Persons with psychotic disorders had the lowest chances to enter paid employment, followed by persons with common mental disorders. Persons with inflammatory conditions had the highest likelihood to enter paid employment. Except for persons with psychotic disorders, the likelihood to enter paid employment was comparable across all age groups. Among persons with psychotic disorders, those aged >45 years had the lowest chance to enter paid employment. The fraction of remaining unemployed due to having psychotic disorders was highest for those aged 45–55 years (PAF 0.19), due to a stronger association between psychotic disorders and entering paid employment (table 3, supplementary table S3)

**Table 3:** Cox proportional hazard ratio (HR) analyses on the influence of chronic diseases on entering paid employment among unemployed persons, stratified by disease and age. [PAF=population attributable fraction]

Chronic diseases (age in years)	N (%)	HR (95% CI)	PAF (95% CI)
Common mental disorders			
18–30	8 385 (11.5)	0.55 (0.52–0.57)	0.09 (0.08–0.09)
30–45	34 703 (17.3)	0.51 (0.50–0.52)	0.14 (0.14–0.15)
45–55	36 446 (20.8)	0.46 (0.45–0.48) ^a^	0.20 (0.19–0.20)
55–65	28 023 (16.4)	0.50 (0.48–0.53)	0.14 (0.13–0.15)
Psychotic disorders			
18–30	3 375 (4.6)	0.41 (0.38–0.43)	0.06 (0.06–0.07)
30–45	12 497 (6.2)	0.29 (0.28–0.31) ^a^	0.13 (0.12–0.14)
45–55	11 082 (6.3)	0.21 (0.20–0.23) ^a^	0.19 (0.18–0.20)
55–65	6 821 (4.0)	0.21 (0.18–0.25) ^a^	0.13 (0.11–0.15)
Cardiovascular diseases			
18–30	2 488 (3.4)	0.82 (0.77–0.87)	0.01 (0.00–0.01)
30–45	15 265 (7.6)	0.75 (0.73–0.77)	0.02 (0.02–0.03)
45–55	35 345 (20.2)	0.75 (0.73–0.76)	0.06 (0.06–0.07)
55–65	59 889 (35.0)	0.74 (0.72–0.76)	0.11 (0.10–0.12)
Diabetes			
18–30	640 (0.9)	0.77 (0.67–0.87)	0.00 (0.00–0.00)
30–45	5 322 (2.7)	0.70 (0.66–0.74)	0.01 (0.01–0.01)
45–55	12 970 (7.4)	0.66 (0.64–0.69) ^a^	0.04 (0.03–0.04)
55–65	21 295 (12.5)	0.68 (0.65–0.71)	0.06 (0.05–0.06)
Inflammatory conditions			
18–30	15 563 (21.4)	0.94 (0.91–0.97)	0.01 (0.01–0.02)
30–45	51234 (25.5)	0.87 (0.86–0.88) ^a^	0.04 (0.03–0.04)
45–55	47 431 (27.1)	0.88 (0.86–0.90) ^a^	0.04 (0.03–0.04)
55–65	39 587 (23.1)	0.96 (0.93–0.99)	0.01 (0.00–0.02)
Respiratory illness			
18–30	4 332 (5.9)	0.89 (0.85–0.94)	0.01 (0.00–0.01)
30–45	17 283 (8.6)	0.79 (0.77–0.81) ^a^	0.02 (0.02–0.03)
45–55	21 635 (12.3)	0.69 (0.67–0.71) ^a^	0.05 (0.05–0.06)
55–65	24 153 (14.1)	0.69 (0.66–0.72) ^a^	0.06 (0.05–0.07)

a Significant difference between age groups (P<0.005), reference group: persons aged 18-30 years.

**Figure 1:**
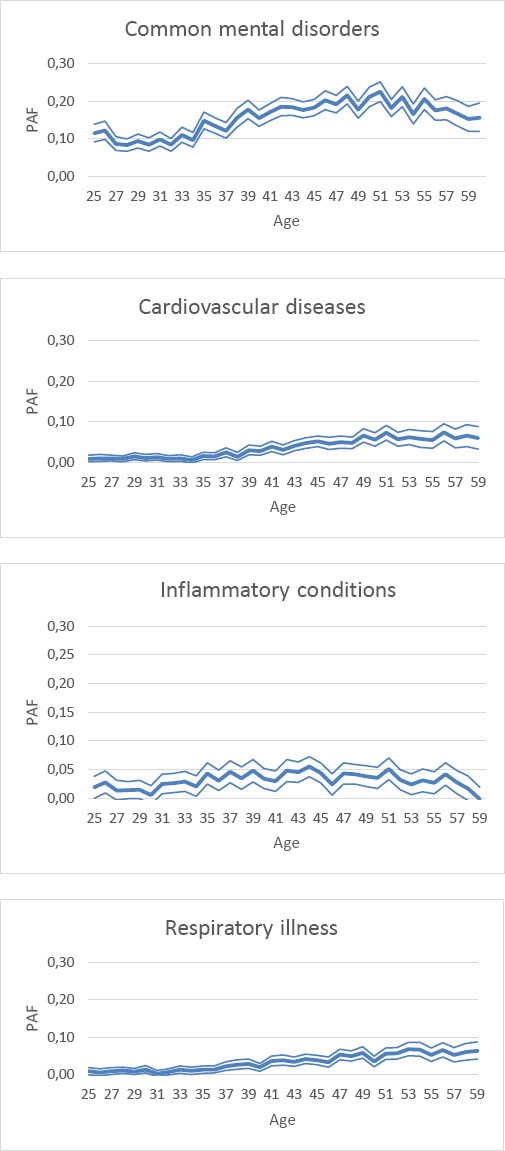
The population attributable fraction (PAF) of four chronic diseases by age.

For persons with common mental disorders, the PAF increased with age to 0.20 until the age of 50 years, and declined onwards to 0.09. The same pattern was observed for inflammatory conditions. The decline in these PAF was primarily caused by a lower prevalence of common mental disorders and inflammatory conditions from middle age onwards. The PAF for cardiovascular diseases increased from 0 at the age of 18 years until 0.10 at the age of 61 years old. The same pattern was observed for respiratory illnesses (figure 1, supplementary figure S1, table 3)

## Discussion

This longitudinal register-based study showed that unemployed persons with chronic diseases were less likely to enter paid employment among all age groups. In particular, persons with psychotic disorders had the lowest likelihood to enter paid employment at all ages, followed by common mental disorders. For psychotic disorders, the impact on maintaining unemployed on population level (PAF 0.20) was primarily due to psychotic disorders being the strongest barrier to enter paid employment. Multimorbidity increased with age, and the impact of having multiple chronic diseases on remaining unemployed increased especially among persons aged ≥45 years. The PAF for common mental disorders and inflammatory conditions increased with age but declined from middle age onwards, whereas the PAF for cardiovascular disorders and respiratory illness gradually increased with age.

The findings of this study corroborate the findings of other studies focusing on the negative influence of poor health on entering paid employment (15, 17, 30, 31). Our study does not suffer from selection bias as the register contains all unemployed persons in The Netherlands. The size of the study population also allowed a detailed insight into the age-specific impact of chronic diseases and multimorbidity on entering paid employment across working age, expressed as the PAF by year of age. The PAF was highest for common mental disorders (PAF 0.20) and psychotic disorders (PAF 0.19) among persons aged 45–55 years. This implies that respectively 20% and 19% of the unemployed persons would have entered paid employment if common mental disorders respectively psychotic disorders were eliminated among this particular age group. This knowledge is particularly valuable for re-integration policies, since targeting persons with common mental disorders or psychotic disorders aged 45–55 years could result in the highest impact on labor force participation at population level compared to other age and disease groups.

This study also adds new insights into the role of chronic diseases and multimorbidity on entering paid employment across different age groups. The current study showed that among younger persons, the presence of one or two chronic diseases had a stronger effect on entering paid employment compared to older persons with one or two chronic diseases. In general, younger persons are more likely to enter paid employment compared to older persons (25). However, our finding can be explained by differences in the type of chronic diseases between younger and older persons. For instance, this study showed that younger persons predominantly suffered from common mental disorders, whereas older persons more often had long-term age-related diseases such as cardiovascular diseases. It may be that age-related diseases such as cardiovascular diseases or diabetes are easier manageable through (modifiable) lifestyle changes and appropriate medication than mental health problems. In this sense, a study by Claussen et al suggested that mental and physical illnesses predict re-employment differently (32, 33).

Many studies investigating the influence of chronic diseases on entering paid employment have used self- reported health measures. Although self-reported diseases may have some advantages, such as being able to include chronic diseases that do not require treatment in healthcare (34, 35), its associated risk of justification bias and reporting bias may be problematic among unemployed persons. To minimize these biases, objective measures of health (eg, register-based data) were used to estimate the effect of chronic conditions on entering paid employment. Moreover, the register contains all unemployed persons in The Netherlands, thus, there is no selection bias. Our results are in line with a Danish register-based study showing that prescription medicine purchases for both physical illnesses (including cardiovascular diseases, chronic lung diseases and type 2 diabetes) and mental illnesses had a negative influence on the likelihood of entering paid employment (21). In accordance with our study, they also found a stronger negative influence (ie, a lower likelihood to enter employment) of mental illnesses on entering paid employment compared to physical health problems such as cardiovascular diseases.

One of the major strengths of this study is the use of objective register-based data. This minimized the risk of reporting and justification bias which is more likely to be present among studies using self-reported health outcomes. In addition, the use of register-based data enabled to include a large study population and thus provided enough statistical power to perform age-specific analyses. Also, use of register-based data allowed a longitudinal design without experiencing challenges such as a low response or loss-to-follow-up, often occurring in other longitudinal studies. Another strength of this study was the calculation of the PAF, which provides insight into the impact of chronic diseases at the population level, combining information on the association of chronic diseases with entering employment and the prevalence of chronic diseases in the population.

The use of register-based data has also limitations. First, registered medication includes merely registrations of individuals who fulfill three criteria: (i) they receive a prescription by their general practitioner or specialist, (ii) they purchase the prescribed medicine at the pharmacy, and (iii) the costs of the medicines are reimbursed by health insurances. It is possible that persons who have certain chronic diseases but are not treated fall outside these criteria. For instance, persons with common mental disorders who are treated with cognitive behavioral therapy will not be covered by registered medication prescriptions. There was no data available on the severity of health conditions. However, health conditions were severe enough for the physician to prescribe medication. A second limitation is that several specific chronic conditions are treated with generic medicines such as paracetamol or other pain-relieving medicines. Therefore, it is not possible to identify specific conditions such as back pain or musculoskeletal disorders.

A third limitation relates to the classification of multimorbidity. The present study operationalized multimorbidity as having at least two different chronic diseases. However, multimorbidity can also be present when an individual suffers from multiple conditions within a particular chronic disease, such as having both rheumatism and eczema as inflammatory conditions. Therefore, it may be that persons with a single chronic disease category may have more conditions and therefore should actually be classified as having multimorbidity. In this study, this limitation may have led to an underestimation of persons with chronic diseases and multimorbidity.

Lastly, the stressful economic and social circumstances of unemployed persons may cause higher utilization of the health care system (36). However, empirical evidence suggests that the higher frequency of visits to a general practitioner among lower educated persons is primarily the result of a higher prevalence of disease rather than earlier care seeking (37). Hence, we do not think that the reported prevalence of medications for chronic diseases, as prescribed by a physician, is influenced by differential care seeking.

The PAF are calculated based on precise estimates of the prevalence of chronic diseases and the association of chronic diseases and entering paid employment using register data on medication and employment status. There are two assumptions underlying a correct interpretation of the PAF (38). First, our longitudinal study design with objective measures of both dependent and independent variables is less sensitive for reversed causality mechanisms and, thus, supports a causal relationship between chronic diseases at baseline and entering paid employment during follow-up. Second, we have adjusted for factors that are known to have a strong influence on the association between chronic diseases and entering employment. However, some other factors may have influenced the likelihood of entering paid employment, such as language skills and specific qualifications. Unfortunately, it was not possible to capture these factors by the registers.

Several recent studies have shown that entering paid employment is associated with improvement of physical and mental health outcomes (39, 40). The positive health effects of paid employment are argued to be mediated by a range of benefits, such as a higher income, having a social role and purpose, and access to and social networks and social support (41, 42). Therefore, there is a need for re-integration policies and interventions that facilitate employment among persons with chronic diseases, particularly among persons with common mental disorders and psychotic disorders. Unemployed persons with chronic diseases may have a reduced work capacity and may experience limitations in performing work (43). It is important that interventions target not only target disease-related factors, but also personal (eg, increasing empowerment and self-management skills) and environmental (eg, facilitating work accommodations) factors that are critical for entering and sustaining employment (44).

It should also be mentioned that stigma in mental health problems may have an impact on employment opportunities for unemployed persons, and their expectations to enter paid employment (45). For instance, studies have shown that employers believed that persons with mental illnesses lack competences to meet the demands of work, and that working is not healthy for them (46, 47). In order to improve employment opportunities for these individuals, the importance of paid employment for persons with mental health problems should be emphasized among clinicians and employers, as it has been shown that employment can contribute to recovery of mental health problems (39).

In conclusion, this register-based study provides detailed insight in the impact of specific chronic diseases on remaining unemployed among different age groups in the population. The age-specific patterns of PAF were highest for mental disorders, due to a high prevalence of common mental disorders, and psychotic disorders, due to a strong association between this chronic disease and remaining unemployed, especially among persons aged 45–55 years old. The impact of multimorbidity on remaining unemployed increased with age. Our results provide directions for interventions and re-integration policies to target chronic diseases among specific age groups in order to reach highest impact on labor force participation at population level.

## Supplementary material

Supplementary material
