# Supplementary material for: The influence of chronic diseases and multimorbidity on entering paid employment among unemployed persons – a longitudinal register-based study
Source: Scand J Work Environ Health. 2021 Mar 31;47(3):208–16. doi: 10.5271/sjweh.3942 (PMC8126442; doi:10.5271/sjweh.3942)
Supplement: Supplementary material [file SJWEH-47-208-S001.pdf]

THE INFLUENCE OF CHRONIC DISEASES AND MULTIMORBIDITY ON ENTERING PAID EMPLOYMENT AMONG UNEMPLOYED PERSONS– A LONGITUDINAL REGISTER-BASED STUDY [1]

Berivan Yildiz, MSc, Alex Burdorf, PhD, Merel Schuring[2], PhD

1. Supplementary material
2. Correspondence to: M. Schuring, Erasmus University Medical Center, Department of Public Health, P.O. Box 2040, 3000 CA Rotterdam, The Netherlands. Email: m.schuring@erasmusmc.nl

Supplementary Table S1. Comparison of the study population of unemployed persons (n=619 968) with persons who were employed (n=7 138 166) on 1st of January 2015.

	**Unemployed**	**Employed**
Age	**n (%)**	**n (%)**
18-30	72 884 (11.8)	1 301 788 (18.2)
30-45	200 829 (32.4)	2 571 643 (36.0)
45-55	175 236 (28.3)	1 975 382 (27.7)
55-65	171 019 (27.6)	1 289 353 (18.1)
Sex		
Male	296 515 (47.8)	3 840 420 (53.8)
Female	323 453 (52.2)	3 297 746 (46.2)
Educational level		
High	92 150 (14.9)	1 924 831 (27.0)
Middle	213 501 (34.4)	1 840 076 (25.8)
Low	279 782 (45.1)	659 098 (9.2)
missing	34 535 (5.6)	2 714 161 (38.0)
Migration background		
Native Dutch	336 916 (54.3)	5 767 332 (80.8)
Moroccan	40 686 (6.6)	98 285 (1.4)
Turkish	32 363 (5.2)	131 181 (1.8)
Surinamese & Antillean	49 221 (7.9)	195 283 (2.7)
Other Western	48 111 (7.8)	557 749 (7.8)
Other non-Western	112 671 (18.2)	388 336 (5.4)
Chronic conditions		
Inflammatory conditions	153 815 (24.8)	1 236 350 (17.3)
Cardiovascular diseases	112 987 (18.2)	787 751 (11.0)
Common mental disorders	107 557 (17.3)	386 567 (5.4)
Respiratory illness	67 403 (10.9)	471 367 (6.6)
Diabetes	40 227 (6.5)	167 281 (2.3)
Psychotic disorders	33 775 (5.4)	45 181 (0.6)
Multimorbidity		
No chronic diseases	253 471 (40.9)	4 302 146 (60.3)
One chronic disease	147 994 (23.9)	1 631 711 (22.9)
Two chronic diseases	93 204 (15.0)	684 594 (9.6)
Three or more chronic diseases	125 299 (20.2)	519 715 (7.3)

Supplementary Table S2. Effect estimates of the interaction of age with entering paid employment among the study population (n=619 968).

	**HR (95% CI)**
Chronic diseases	
0	1
1	0.81 (0.79-0.83)
2	0.58 (0.56-0.60)
≥3	0.29 (0.28-0.30)
Age	
18-30	1
30-45	0.66 (0.63-0.68)
45-55	0.54 (0.52-0.56)
55-65	0.30 (0.29-0.31)
Interaction terms	
1 chronic disease * 18-30	1
1 chronic disease * 30-45	0.92 (0.89-0.95)
1 chronic disease * 45-55	0.93 (0.90-0.96)
1 chronic disease * 55-65	1.03 (0.99-1.07)
2 chronic diseases * 18-30	1
2 chronic diseases * 30-45	0.82 (0.79-0.86)
2 chronic diseases * 45-55	0.84 (0.80-0.88)
2 chronic diseases * 55-65	0.98 (0.93-1.03)
≥3 chronic diseases * 18-30	1
≥3 chronic diseases * 30-45	0.64 (0.60-0.68)
≥3 chronic diseases * 45-55	0.57 (0.54-0.61)
≥3 chronic diseases* 55-65	0.68 (0.64-0.72)

Supplementary Table S3. Effect estimates of interaction term analyses of age with entering paid employment in the study population (n=619 968).

	**Common mental disorders**	**Psychotic disorders**	**Cardiovascular diseases**	**Diabetes**	**Inflammatory conditions**	**Respiratory illness**
No	1	1	1	1	1	1
Yes	0.47 (0.46-0.49)	0.18 (0.16-0.19)	0.73 (0.71-0.76)	0.63 (0.58-0.68)	0.88 (0.86-0.91)	0.66 (0.64-0.69)
Age						
18-30	ref	Ref	ref	ref	ref	ref
30-45	0.79 (0.76-0.81)	0.67 (0.65-0.70)	0.77 (0.74-0.80)	0.75 (0.70-0.80)	0.77 (0.76-0.80)	0.75 (0.73-0.78)
45-55	0.56 (0.55-0.57)	0.42 (0.40-0.44)	0.58 (0.56-0.60)	0.53 (0.50-0.57)	0.56 (0.55-0.57)	0.53 (0.51-0.54)
55-65	0.23 (0.22-0.24)	0.17 (0.15-0.18)	0.25 (0.24-0.26)	0.23 (0.21-0.25)	0.24 (0.23-0.24)	0.22 (0.21-0.22)
Disease*18-30	ref	ref	ref	ref	ref	ref
Disease*30-45	0.96 (0.92-1.01)	0.72 (0.66-0.78)	0.95 (0.89-1.02)	0.90 (0.79-1.04)	0.94 (0.91-0.97)	0.91 (0.87-0.97)
Disease*45-55	0.89 (0.85-0.93)	0.52 (0.47-0.57)	0.96 (0.90-1.02)	0.84 (0.73-0.96)	0.95 (0.92-0.98)	0.82 (0.77-0.86)
Disease*55-65	0.94 (0.89-1.00)	0.51 (0.44-0.60)	0.97 (0.91-1.04)	0.90 (0.78-1.03)	1.03 (0.99-1.08)	0.80 (0.75-0.86)

Supplementary Table S4. Description of ATC-codes used to identify chronic diseases among the study population (N=619 968).

Chronic disease	ATC-code	Medication class
**Cardiovascular diseases**	B01A<br>C01<br>C03A<br>C08<br>C07<br>C09A, C09B	Antithrombotic agents<br>Cardiac agents<br>Low-ceiling drugs<br>Calcium channel blockers<br>Beta blocking agents<br>ACE inhibitors
**Common mental disorders (anxiety, depression, sleep disorders)**	N05B<br>N05C<br>N06A	Anxiolytics<br>Hypnotics and sedatives<br>Antidepressants
**Inflammatory conditions**	M01A	Anti-inflammatory and antirheumatic products, non-steroids
**Respiratory illness**	R03A, R03C<br>R03B, R03D	Adrenergics (inhalants)<br>Other drugs for obstructive airway diseases
**Psychotic disorders (psychoses)**	N05A	Antipsychotics
**Diabetes mellitus**	A10A<br>A10B	Insulins and analogues<br>Blood glucose lowering drugs

* Chronic diseases were identified using the ATC-codes of prescribed medication following the study of Huber on identification of chronic diseases by ATC codes (1) and the study of Van Ooijen (2).
** We were able to identify many other chronic diseases such as cancer and dementia, based on the registered medicines used for the treatment of these diseases. Our measure of multimorbidity includes 21 chronic diseases as identified by the study of Huber. Regarding specific chronic diseases in our study, we only investigated chronic diseases with a prevalence higher than 5% in our study population. Therefore, diseases with a prevalence lower than 5% in our study population (such as cancer) were not investigated as specific chronic disease. In addition, also diseases that usually do not required prescription of medication are not included in our study.

Supplementary Figure S1. The prevalence and Hazard Ratio of chronic diseases at each age among unemployed persons.

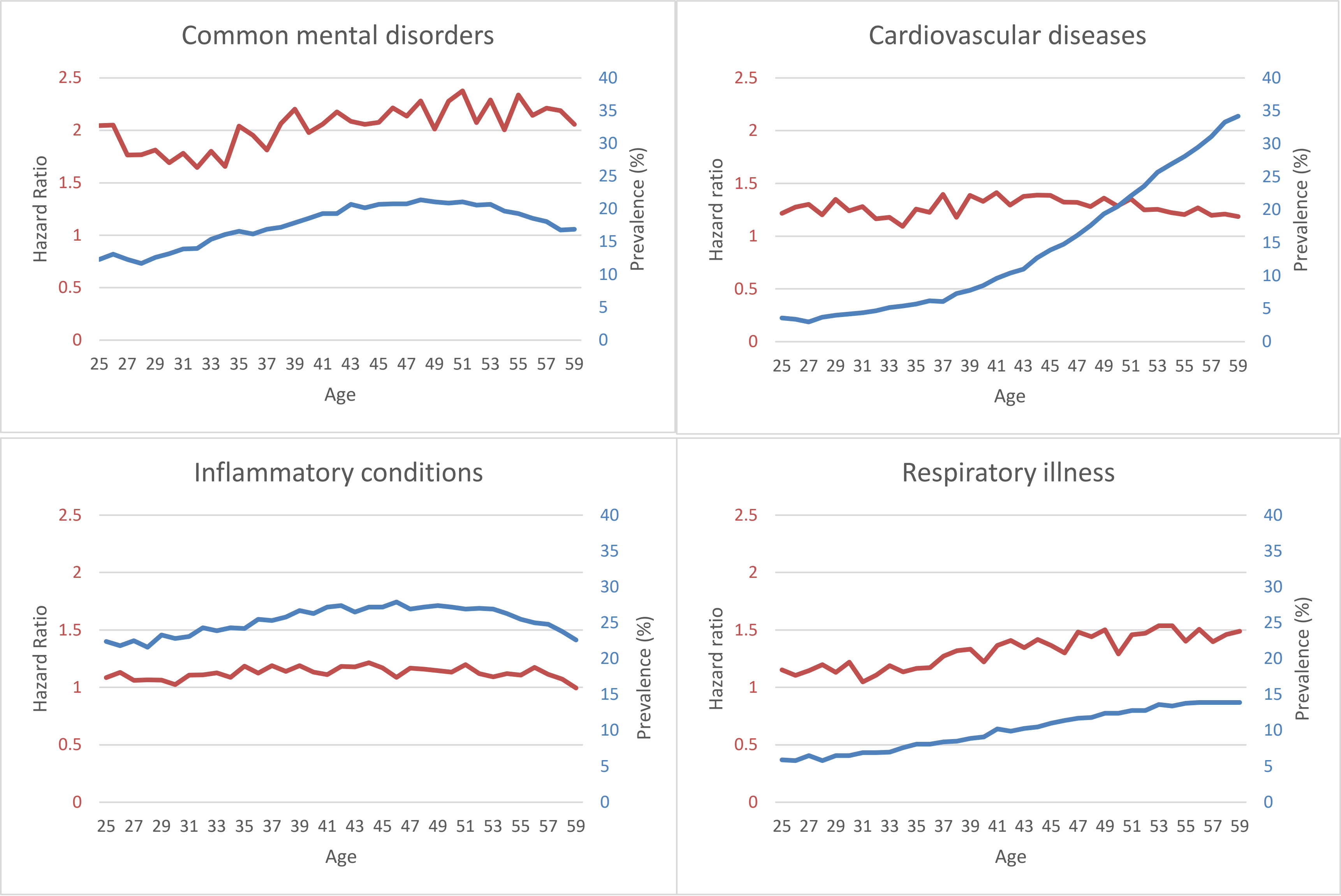

1. Huber CA, Szucs TD, Rapold R, Reich O. Identifying patients with chronic conditions using pharmacy data in Switzerland: an updated mapping approach to the classification of medications. BMC Public Health. 2013;13:1030-.
2. van Ooijen R. Life cycle behavior under uncertainty: Essays on savings, mortgages and health. Groningen: University of Groningen, SOM research school; 2016.
